# α-Mangostin reduces hypertension in spontaneously hypertensive rats and inhibits EMT and fibrosis in Ang II-induced HK-2 cells

**DOI:** 10.7150/ijms.94236

**Published:** 2024-06-17

**Authors:** Yuhui Xu, Jianhua Wu, Lihui Gao, Hua Lin, Zhiqiang Yang, Xiao Liu, Yanfen Niu

**Affiliations:** 1 School of Basic Medical Sciences, Kunming Medical University, Kunming 650500, China.; 2 Science and Technology Achievement Incubation Center, Kunming Medical University, Kunming 650500, China.

**Keywords:** α-Mangostin, hypertension, hypertensive nephropathy, epithelial-to-mesenchymal transformation, TGF-β signaling pathway.

## Abstract

Hypertension affects a large number of individuals globally and is a common cause of nephropathy, stroke, ischaemic heart disease and other vascular diseases. While many anti-hypertensive medications are used safely and effectively in clinic practice, controlling hypertensive complications solely by reducing blood pressure (BP) can be challenging. α-Mangostin, a xanthone molecule extracted from the pericarp of *Garcinia mangostana L*., has shown various beneficial effects such as anti-tumor, anti-hyperuricemia, and anti-inflammatory properties. However, the effects of α-Mangostin on hypertension remain unknown. In this study, we observed that α-Mangostin significantly decreased systolic and diastolic blood pressure in spontaneously hypertensive rats (SHR), possibly through the down-regulation of angiotensin II (Ang II). We also identified early markers of hypertensive nephropathy, including urinary N-acetyl-β-D-glucosaminidase (NAG) and β2-microglobulin (β2-MG), which were reduced by α-Mangostin treatment. Mechanistic studies suggested that α-Mangostin may inhibit renal tubular epithelial-to-mesenchymal transformation (EMT) by down-regulating the TGF-β signaling pathway, thus potentially offering a new therapeutic approach for hypertension and hypertensive nephropathy.

## Introduction

Recent studies have shown that approximately one-third of adults worldwide suffer from hypertension, and it is estimated that 8.5 million deaths are attributable to hypertension or hypertension related diseases, such as nephropathy, stroke, ischaemic heart disease and other vascular diseases^1^, ^2^. About 90%-95% patients with hypertension have primary hypertension. Several risk factors associated with primary hypertension include age, sex, genetic, overweight, diabetes mellitus, smoking, excess intake of sodium and alcohol^2^. Most of these risk factors, such as age, sex, sodium consumption and genetic, have important effects on Angiotensin II (Ang II). Ang II binds to the type 1 receptor (AT1R) and induces hypertension by regulating vasoconstriction, stimulating the sympathetic nervous system, activation sodium and water retention and promoting aldosterone secretion^3^-^6^. On the other hand, activation of the type 2 receptor (AT2R) by Ang II is beneficial to hypertensive patients, including vasodilation via release of bradykinin and nitric oxide, anti-inflammation, increasing renal blood flow, promoting natriuresis and improving heart damage^7^, ^8^.

Currently, there are numerous safe and effective anti-hypertension medications available for clinical use. However, the effecacy of drugs used to treat complications of hypertensive, particularly in the management of hypertensive nephropathy, is often limited. Hypertensive patients frequently experience varying degrees of kidney damage, such as inflammation, fibrosis, glomerulosclerosis, vascular intimal hyperplasia, and other renal conditions^9^, ^10^. Continuous kidney damage can lead to the progression of end-stage renal disease^3^.

Ang II is a key factor that induces renal tubular epithelial-mesenchymal transition (EMT) and fibrosis by activating the TGF-β signaling pathway^11^. Tubular EMT and subsequent fibrosis are early events that contribute to the development of nephropathy^12^, ^13^. Studies have shown that inhibition of the TGF-β-Smad3 pathway by the Ang II receptor inhibitor (AT_1_R) losartan significantly reduces renal tubule fibrosis in hypertensive^14^, ^15^. Additionally, kidney damage in Ang II-induced mice was effectively alleviated by inhibiting TGF-β signaling with MiR-101a^16^. Therefore, down-regulating the TGF-β-Smad pathway to inhibit EMT-fibrosis of renal tubular epithelium may be a promising strategy for the treatment of hypertensive nephropathy.

Natural products are valuable resources for drug discovery. *Garcinia mangostana L.,* a common subtropical fruit found in Malaysia, Indonesia, Thailand and southern China, has been traditionally used as herbal medicine for treating various ailments such as abdominal pain, dysentery, diarrhea, suppuration and chronic ulcers^17^. α-Mangostin, a xanthone compound abundant in the pericarp of *Garcinia mangostana L*., has gained attention for its beneficial effects on diseases such as tumor, hyperuricemia, inflammation, and metabolic disorders 18-20. However, the effects of α-Mangostin on hypertension remain unclear. This study aims to investigate the therapeutic effect and mechanisms of α-Mangostin on hypertension and associated kidney damage in spontaneously hypertensive rats (SHR).

## Materials and Methods

**Compounds** α-Mangostin used in this study is the same monomeric compound as in our previous study^19^. Losartan was purchased from Huahai Pharmaceutical Co., Ltd, (Zhejiang, China); Batch No: 0000012720.

**Animal Treatment** Rats were housed and handled in accordance with the guidelines of the Institutional Animal Care and Use Committee. Every effort was made to minimize the use and suffering of the animals. Twelve-week-old male spontaneously hypertensive rats (SHR) and Wistar Kyoto (WKY) rats (SCXK 2019-0010) were obtained from SPF Biotechnology Co., Ltd. (Beijing, China) and kept in sterile facilities at a controlled temperature of 25 °C, with ad libitum access to water and food. SHR were divided into model, losartan (20.0 mg/kg) and α-Mangostin (0.5 and 1.0 mg/kg) groups, while WKY rats were used as control group.

Rats were administered vehicle (0.5% Sodium carboxymethyl cellulose), α-Mangostin (0.5 or 1 mg/kg/day), or losartan (20 mg/kg/day) intragastrically (i.g.) for 6 weeks. Blood pressure was measured using the BP-2000 SERIES II Blood Pressure Analysis System every week after 1 hour of treatment. Rats were anesthetized with pentobarbital sodium at dose of 50 mg/kg. Renal tissues and serum were collected after 1 hour after the final administration and stored at -80 °C for further analysis. Urine was collected for 3 hours using metabolism cages at the 5^th^ week after administration.

**Biochemical Marker and ELISA Analysis** The blood was collected from the abdominal aorta and centrifuged at 2300 *rcf* for 10 min to obtain serum. Urine microalbumin (mAlb) and β2-microglobulin (β2-MG), as well as serum Ang II levels, were detected using commercial rat ELISA kits (Jiangsu Enzyme-linked Biotechnology Co., Ltd., China), following with the specified protocol. Urine creatinine (Cr) and N-Acetyl-β-D-Aminoglucosidase (NAG) were measured using a commercially available kit (Nanjing Jiancheng Bioengineering Research Institute, China).

**Cell Culture and Treatment** HK-2 cells (Human renal tubular epithelial cells) were purchased from American Type Culture Collection (Manzas, Virginia) and cultured in DMEM-F12 medium supplemented with 10% fetal bovine serum (FBS), 100 units/ml penicillin, and 100 mg/ml streptomycin in a humidified atmosphere of 95% air and 5% CO_2_ at 37 °C. The cells were passaged continuously by trypsinization of subconfluent cultures and the medium was replaced every 2 days.

HK-2 cells were seeded on 96-well or 6-well pates using low-glucose medium when reaching 70% - 80% confluency. Once cells reached 90% confluency, they were maintained in FBS-free low-glucose medium for 12 hours, followed by incubation with Ang II (10^-7^ mol/L), losartan (5×10^-6^ mol/L), and α-Mangostin (10^-9^∼10^-7^mol/L) for 24 or 48 hours. If the incubation was 48 hours, the medium or drugs were changed every 24 hours.

**Western Blotting Analysis** RIPA lysis buffers were used to lyse HK-2 cells by incubating them on ice for 10 min. The total protein levels of supernatants were determined using a commercial BCA protein assay kit (CoWin Biotech Co., Ltd.). Samples were separated by 10% SDS-PAGE gels and transferred to PVDF membranes (IPVH00010, Millipore). After incubating with 5% skim milk for 1 hour at room temperature, the membranes were incubated with different diluted antibodies, including E-cadherin (Proteintech, 20874-1-AP, 1:2000), Vimentin (Proteintech, 10366-1-AP, 1:1000), α-SMA (CST, 19245S, 1:1000), GAPDH (Proteintech, 10017731, 1:50000), Smad2 (ABclonal A11498, 1:1000), p-Smad2 (ABclonal AP0909, 1:1000), Smad3 (ABclonal A11388, 1:2000), TGF-β-1 (Origene, D925AA011, 1:2000) and TGF-β1R (Wanleibio, WL03150, 1:2000) overnight at 4 °C. Then, the membranes were incubated with secondary diluted anti-rabbit IgG (202700804, Zhongshanjinqiao, Beijing, China, 1:8000), or anti-mouse IgG antibodies (224800105, Zhongshanjinqiao, Beijing, China, 1:8000) in 3% skim milk for 1 to 2 hours at room temperature. PVDF membranes were incubated with HRP substrate and detected using a BIO-RAD chemiluminescence Imager.

**Real-Time PCR** Total RNA was extracted from HK-2 cells using TRIzol® (Q5704, Tiangen, China). cDNA was synthesized following the manufacturer's protocol (00425005, ThermoFisher). mRNA levels were determined using the SYBR Ⓡ FAST Real-time PCR Kit (0000095770, KAPA) and analyzed using the 2^-ΔΔCt^ method. The primer sequences for real-time PCR are listed in **Table 1**. GAPDH was utilized as a housekeeping gene.

**HK-2 Cells Wound Healing Assay** HK-2 cells were seeded in 6-well pates and treated as described above. The cells were scratched using a 200-µl pipette tip. Images were captured at the initial time point and 48 hours later using microscopy at the same location. Wound closure was analyzed with Image-Pro-Plus6.0 software, and the migration rate (%) was calculated as [(0^th^ area - 48^th^ area) ×100% / 0^th^ area].

**Statistical Analysis** All data were presented as mean ± standard deviation. Each cell experiment was repeated at least three times. One-way analysis of variance (ANOVA) was used for multiple-group comparisons. If normal distribution was confirmed by *F* test, an independent two-tailed Student's* t* test was conducted between two groups; otherwise, Satterthwaite's *t* test was used. Statistical significance was considered at* P* < 0.05.

## Results

### α-Mangostin reduced systolic and diastolic blood pressure as well as serum Ang II levels in Spontaneously Hypertensive Rats (SHR)

**Figure 1:**
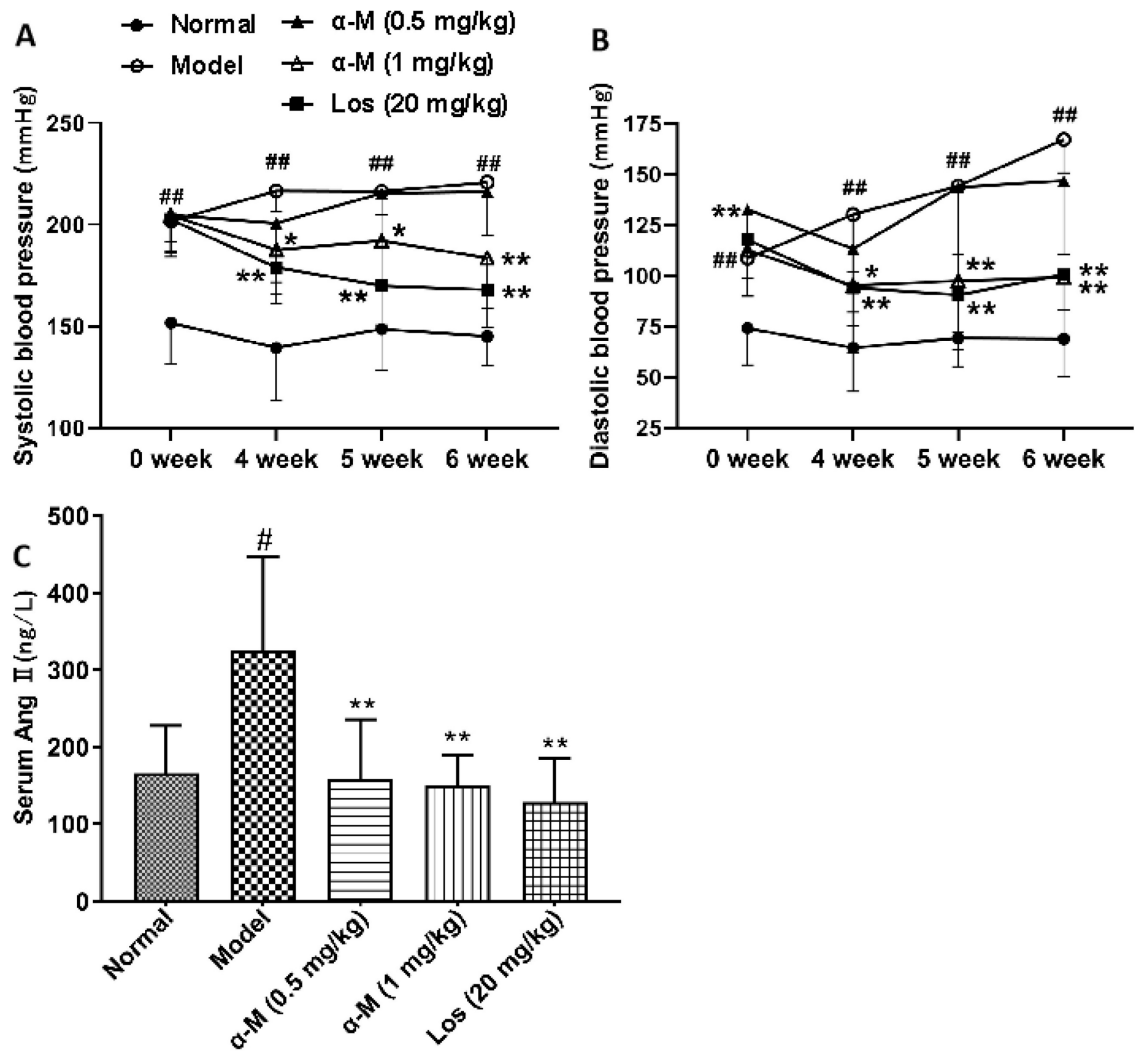
SHR were treated with either vehicle (0.5 % CMC-Na), α-Mangostin (0.5 or 1 mg/kg), or Losartan (20 mg/kg) once a day for 6 weeks. Blood pressure was measured 1 hour after administration at the 4^th^, 5^th^ and 6^th^ weeks. Error bars represent the standard deviation (SD), with n = 6 - 10. ^*^P < 0.05 and ^**^P < 0.01 compared to the Model.*^##^P* < 0.01 compared to the Normal. Normal refers to Wistar rat, Model refers to SHR, α-M refers to α-Mangostin, and Los refers to Losartan.

Treatment with α-Mangostin (1 mg/kg) effectively lowered systolic and diastolic blood pressure in SHR at the 4^th^, 5^th^ and 6^th^ weeks **(Fig. 1A and B)**. The anti-hypertensive effect of α-Mangostin (1 mg/kg) was comparable to that of losartan (20 mg/kg). Furthermore, serum Ang II levels were significantly decreased after treatment with α-Mangostin or losartan, suggesting that the anti-hypertensive mechanism of α-Mangostin may be related to the reduction of Ang II **(Fig. 1C)**. These findings indicate that α-Mangostin could be a promising agent for the treatment of hypertension.

### α-Mangostin reduced early kidney damage markers in SHR

The kidney is highly affected by hypertension, but current treatment for hypertensive nephropathy have not shown optimal efficacy. As shown in **Table 2**, there was no significant change in urinary Cr and mAlb levels, while urinary NAG and β2-MG levels were significantly elevated in SHR. Importantly, treatment with α-Mangostin resulted in a significant reduction in urinary levels of NAG and β2-MG, which are sensitive markers of early renal damage, suggesting potential renoprotective effects in SHR.

### α-Mangostin prevented Ang II-induced migration in HK-2 cells

**Figure 2:**
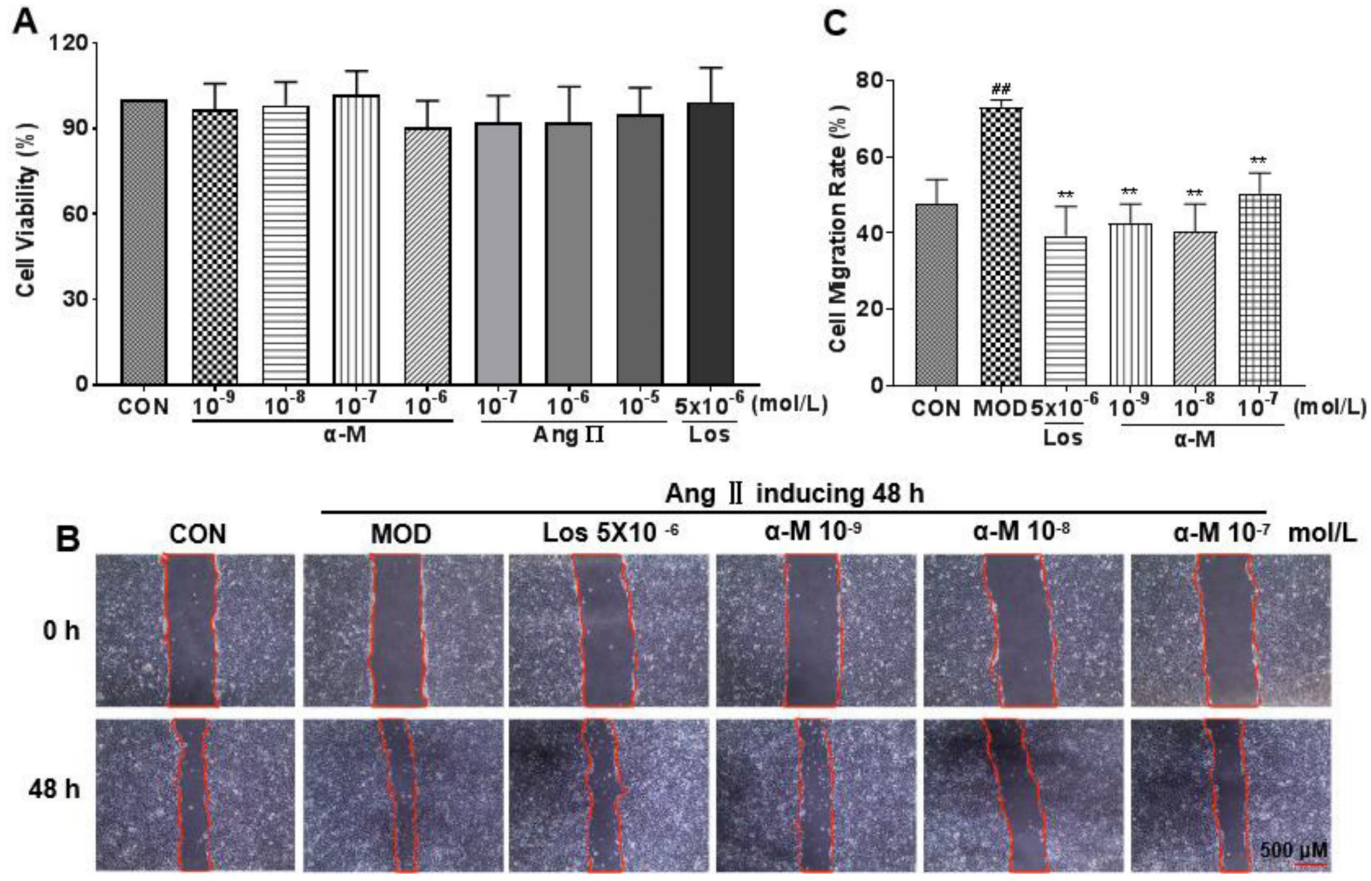
α-Mangostin inhibited Ang II-induced migration in HK-2 cells. **(A)** HK-2 cells were treated by different drugs for 48 hours, and the cell numbers were detected using CCK-8 kit. **(B)** Migration of HK-2 cells after treatment with α-Mangostin or Losartan for 48 hours. Scale bars represent 500 μm (40 ×). **(C)** Quantified migration rate of **(B)**. Error bars represent the SD; ^**^P < 0.01 compared to the MOD group; *^##^P* < 0.01 compared to the CON group; n = 3. Los, Losartan; α-M, α-Mangostin.

Epithelial-mesenchymal transition (EMT) of renal tubular epithelial cells is the early stage of renal damage, and cell migration is a key step for renal tubular epithelium undergoing EMT and subsequent fibrosis. As shown in CCK-8 (Cell Counting Kit-8) experiment (**Fig. 2A**), cell proliferation was not affected at different concentrations. The concentrations of Ang II and losartan used *in vitro* experiments were 10^-7^ mol/L and 5×10^-6^ mol/L, respectively. Cell migration rate was significantly increased by Ang II induction; however, it was markedly inhibited after α-Mangostin and losartan treatment for 48 hours (**Fig. 2B and C**). These results provide evidence that EMT in HK-2 cells may be inhibited by α-Mangostin.

### α-Mangostin inhibited the EMT markers in Ang II-induced HK-2 cells

**Figure 3:**
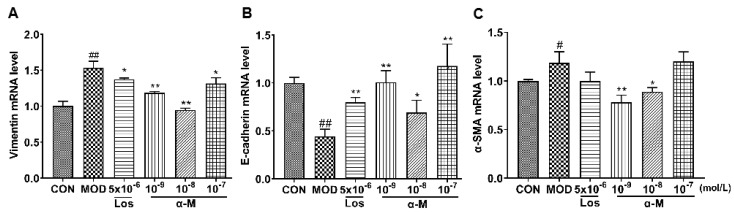
α-Mangostin rescued the mRNA levels of EMT markers in Ang II-induced HK-2 cell. GAPDH was used as the control. **(A-C)** The mRNA levels of vimentin, E-cadherin, and α-SMA were measured after 24 hours of treatment with vehicle, α-Mangostin or Losartan. Error bars represent the SD; ^**^P < 0.01, ^*^P < 0.1, compared to the MOD group; *^##^P* < 0.01, *^#^P* < 0.1, compared to the CON group; n = 3. Los: Losartan; α-M: α-Mangostin.

**Figure 4:**
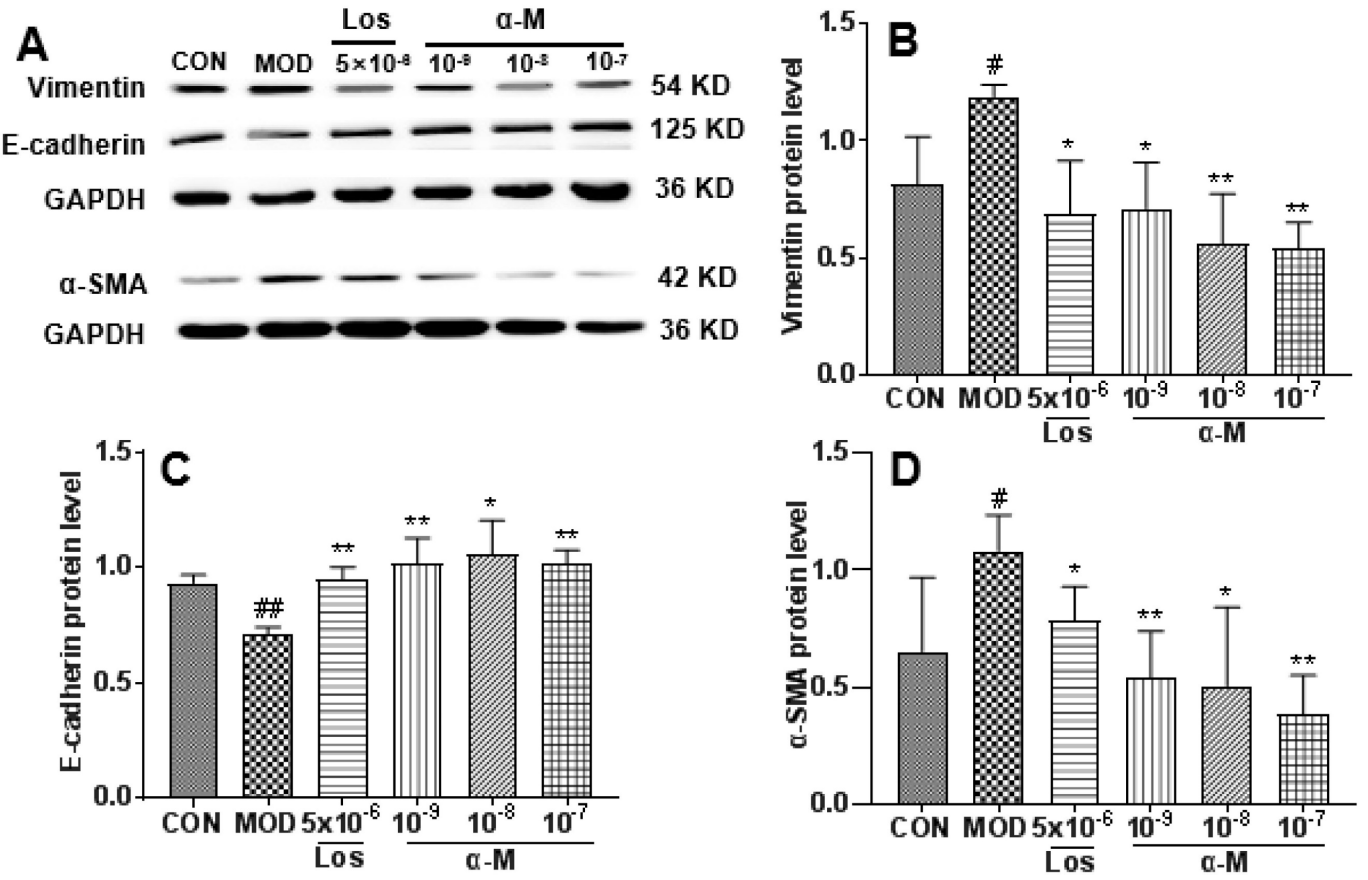
α-Mangostin rescued the protein levels of EMT markers in Ang II-induced HK-2 cells, with GAPDH used as the control. **(B-D)** Protein levels of vimentin, E-cadherin, and α-SMA were assessed after 24 hours of treatment. Error bars indicate the SD; ^**^P < 0.01, ^*^P < 0.1, compared to the MOD group; *^##^P* < 0.01, *^#^P* < 0.1, compared to the CON group; n = 3 - 4. Los: Losartan; α-M: α-Mangostin.

E-cadherin, a crucial protein that inhibits EMT progression by enhancing the adherence and junction of epithelial cells, was significantly increased after treatment with α-Mangostin and losartan, while the expression of α-SMA and vimentin, which are markers of mesenchymal and fibrotic cells associated with EMT progression, was markedly decreased, as demonstrated in Fig. 3 and Fig. 4. These results indicate that α-Mangostin effectively suppressed EMT progression in Ang II-induced HK-2 cells by modulating EMT markers.

### α-Mangostin suppressed the EMT progression by inhibiting the TGF-β1-smad signaling pathway in Ang II-induced HK-2 cells

**Figure 5:**
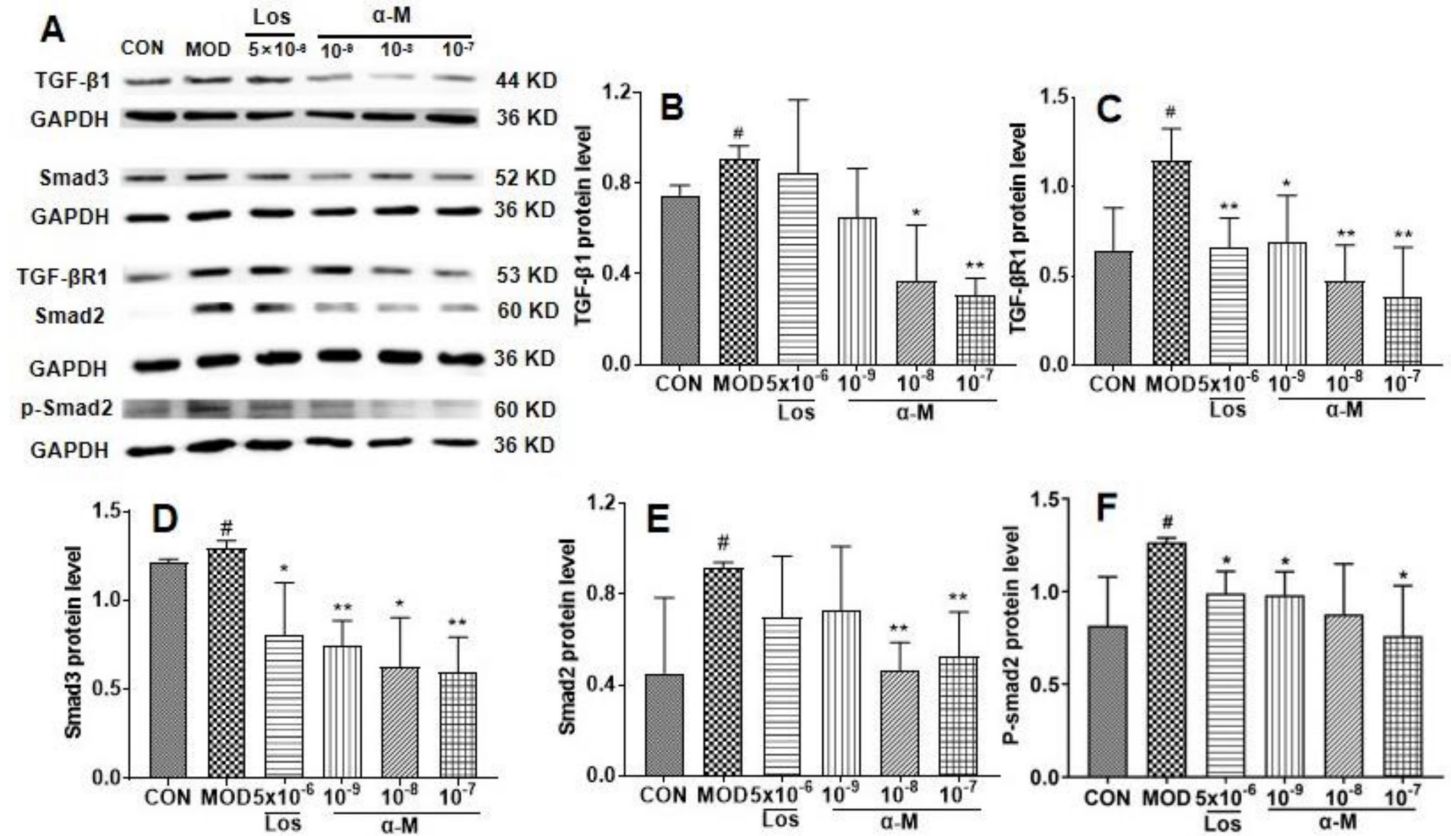
α-Mangostin inhibited the TGF-β signaling pathway in Ang II-induced HK-2 cells. **(B-F)** The protein levels of TGF-β1, TGF-β1R, Smad3, Smad2, and p-Smad2 in Ang II-induced HK-2 cells were assessed after 24 hours of treatment. GAPDH was used as the control. Error bars indicate the SD. ^**^P < 0.01, ^*^P < 0.1, compared to the MOD group; *^##^P* < 0.01, *^#^P* < 0.1, compared to the CON group; n = 3 - 4. Los: Losartan; α-M: α-Mangostin.

Previous studies have demonstrated that the activation of the TGF-β-smad3 pathway is crucial for Ang II-induced renal tubular epithelial EMT and fibrosis^21^, ^22^. In this study**,** α-Mangostin significantly decreased Ang II levels in SHR serum. Therefore, we investigated the effects of α-Mangostin on the TGF-β1 pathway. As shown in **Fig. 5**, the protein levels of TGF-β1, TGF-β1R, and their downstream Smad2/3 and p-smad2 were significantly inhibited by α-Mangostin treatment. These results suggested that α-Mangostin may suppress EMT in HK-2 cells by inhibiting the TGF-β1-Smad signaling pathway.

## Discussion

In clinical practice, angiotensin-converting enzyme inhibitors (ACEI), Ang II receptor blockers (ARBs) and calcium channel blockers (CCBs) are commonly used for treating hypertensive patients with kidney damage. These drugs have shown beneficial effects in the kidney protection by reducing albuminuria and slowing the progression of nephropathy^23^. However, they may not always achieve optimal therapeutic outcomes due to poor efficacy and side effects such as cough, angioedema, taste disturbances, and hyperkalemia^23^, ^24^. α-Mangostin, extracted from the traditional medicinal herb *Garcinia mangostana L.*, has demonstrated significant improvements in hypertension and the epithelial-mesenchymal transition (EMT) of HK-2 cells. It may hold promise as a potential agent for the treatment of hypertension and nephropathy.

Urine creatinine (Cr) and microalbumin (mAlb) are important markers of renal function, typically increasing when the kidneys are undergoing damages. Urine NAG and β2-MG are sensitive markers indicating renal tubule injury, even showing increases when the renal tubules are only slightly impaired in the early stages^25^, ^26^. In this study, levels of urine NAG and β2-MG significantly decreased after treatment with α-Mangostin, suggesting that α-Mangostin may protect against early renal damage in SHR. However, further investigation into the effects of α-Mangostin on hypertensive nephropathy in its end stages is needed.

EMT is one of important sign in the early stage of renal tubular damage, which is an important pathological process in hypertensive nephropathy^12^, ^27^. During EMT, the expression of E-cadherin decreases, leading to the progressive loss of adhesion and polarity in epithelial cells, allowing them to acquire migratory ability and move into the interstitial space. α-SMA and vimentin, synthesized by mesenchymal and fibrotic cells, increase as the cells migrate to the mesenchymal space and transform into mesenchymal cells.^28^. Subsequently, mesenchymal cells differentiate into myofibroblasts, ultimately resulting in tubulointerstitial fibrosis.^29^. The loss of renal tubule epithelial cells leads to the leakage of small molecular proteins, promoting the formation of microalbuminuria. Interstitial fibrotic cells surround the peritubular capillaries, eventually causing capillary hyalinosis and glomerulosclerosis^28^. Inhibiting renal tubular EMT-fibrosis may be a promising strategy for preventing and treating hypertensive nephropathy.^27^. The study showed that the markers of migration and EMT were significantly inhibited in Ang II induced HK-2 cells, suggesting that α-Mangostin may be a potential agent for treating hypertensive nephropathy.

Previous studies have identified numerous beneficial effects of α-Mangostin, such as its anti-hyperlipidemic, anti-hyperuricemic, anti-inflammatory, and anti-hyperglycemic properties.^18^, ^19^, ^30^. However, the specific target of α-Mangostin has not been elucidated. In this study, α-Mangostin did not exhibit a dose-dependent effect, possibly due to its complex mechanism of regulating multiple signaling pathways. Additionally, hypertensive patients often suffer from multiple metabolic diseases, including hyperlipidemia, hyperglycemia, and hyperuricemia. Multi-effects drug, like metformin, have shown significant therapeutic advantages in treating metabolic diseases. Therefore, α-Mangostin may be a promising agent for treating hypertensive patients with co-morbid metabolic diseases.

Previous studies have demonstrated that the activation of the TGF-β-smad3 signaling pathway is crucial for Ang II-induced renal tubular epithelial EMT and fibrosis^21^, ^22^. Correspondingly, inhibition of the TGF-β pathway results in significant improvement of hypertensive nephropathy by suppressing renal tubular epithelial EMT and fibrosis^14^, ^16^. In our study, treatment with α-Mangostin inhibited the TGF-β1-smad3 pathway in Ang II-induced HK-2 cells, which is consistent with previous findings showing that α-Mangostin attenuates cardiac and liver fibrosis by inhibiting TGF-β-Smads pathway 31, 32.

In other studies, the oral dosage of α-Mangostin in rats ranged from 25 to 200 mg/kg^31^, ^33^. In this study, the effective doses of α-Mangostin were 0.5 and 1 mg/kg. It is worth noting that in our previous study, α-Mangostin exhibited significant effects on hyperuricemia in mice at a dosage of 1 mg/kg^19^. The efficacy and pharmacokinetics associated with different concentrations of α-Mangostin warrant further investigation.

In conclusion, hypertension affects a large number of individuals worldwide, with hypertensive nephropathy being the most common cause of end-stage renal disease and difficult to prevent^34^. Recent studies have shown that renal tubular EMT is a key pathological process in hypertensive nephropathy^12^, ^27^. This study is the first to demonstrate the beneficial effects of α-Mangostin in reducing blood pressure and early renal damage in spontaneously hypertensive rats (SHR). The renoprotective effects of α-Mangostin may be attributed to its ability to inhibit renal tubular EMT through the down-regulation of TGF-β1 signaling. α-Mangostin could potentially serve as a therapeutic agent for the treatment of hypertension and hypertensive nephropathy.

## Figures and Tables

**Table 1:** Primer sequences used in the study.

Genes	Primer Sequences
Human E-cadherin	For: 5'-CGTCGTAATCACCACACTGAAA-3' Rev: 5'-GTAGCAACTGGAGAACGTTATTT-3'
Human Vimentin	For: 5'-TGCGTCTCTGGCACGTCTTG-3' Rev: 5'-GGACATGCTGTTCCTGAATCTG-3
Human α-SMA	For: 5'-GAGAAGAGTTACGAGTTGCCTGA-3' Rev: 5'-GATGCTGTTGTAGGTGGTTTCA-3'
Human GAPDH	For: 5'-GGAAGGAAATGAATGGGCAGC-3' Rev: 5'-AGTTAAAAGCAGCCCTGGTGA-3'

**Table 2:** α-Mangostin reduced urine NAG and β2-MG in SHR.

Groups	Dosage (mg/kg)	Cr (mmol/L)	mAlb (μg/L)	NAG (U/L)	β2-MG (μg/L)
Normal	Vehicle	5.5±1.9	313.3±32.2	10.6±6.9	35.7±2.1
Model	Vehicle	6.5±1.1	297.9±36.5	43.1±17.8^##^	55.5±6.5^##^
α-Mangostin	0.5	6.4±1.8	300.8±28.7	15.6±14.9^*^	36.8±3.9^**^
	1	7.7±3.1	291.2±45.6	30.7±11.2	38±9.4^**^
Losartan	20	5.9±2.5	275.2±25.0	19.0±16.5	41.6±10.1^*^

**Table 2.** Urine was collected for 3 hours after administration at the 5^th^ week. Error bars represent the SD, n = 5 - 8. ^*^P < 0.05 and ^**^P < 0.01 compared to Model.*^##^P* < 0.01 compared to Normal.
